# The involvement of cyclotides in the heavy metal tolerance of *Viola* spp.

**DOI:** 10.1038/s41598-024-69018-x

**Published:** 2024-08-20

**Authors:** Klaudia Sychta, Aneta Słomka, Reza Shariatgorji, Per E. Andrén, Sławomir Samardakiewicz, Ulf Göransson, Blazej Slazak

**Affiliations:** 1 Department of Plant Cytology and Embryology, Institute of Botany, Faculty of Biology, Jagiellonian University in Kraków, 9 Gronostajowa St, 30-387 Cracow, Poland; 2 W. Szafer Institute of Botany of the Polish Academy of Sciences, 46 Lubicz, 31-512 Krakow, Poland; 3 Department of Pharmaceutical Biosciences, Uppsala University, P.O. Box 574, 751 23 Uppsala, Sweden; 4 Department of Pharmaceutical Biosciences, Science for Life Laboratory, Spatial Mass Spectrometry, Uppsala University, P.O. Box 591, 751 24 Uppsala, Sweden; 5 Laboratory of Electron and Confocal Microscopy, Faculty of Biology, Adam Mickiewicz University, 6 Uniwersytetu Poznańskiego St, 61-614 Poznań, Poland

**Keywords:** Cyclotides, *Viola*, Heavy metals, Cell suspension culture, Biotechnology, Cell biology, Chemical biology, Molecular biology, Plant sciences, Environmental sciences

## Abstract

The Violaceae family is rich in metal-tolerant species and species producing cyclic peptides (cyclotides) that are linked to the resistance to biotic factors. Plants that inhabit areas polluted with heavy metals have developed various mechanisms of tolerance. To test the role of cyclotides in protection against abiotic factors, including heavy metals, cell suspension cultures of *Viola* species/genotypes (*V. lutea* ssp. *westfalica*, *V. tricolor*, *V. arvensis,* and *V. uliginosa*), representing different levels of tolerance to heavy metals (from the most tolerant-MET to the least tolerant populations/species-NMET), were used. The relative abundances of the cyclotides in the control, untreated cell suspensions of all the selected species/genotypes, and cells treated with Zn or Pb (200 µM or 2000 µM) for 24 h or 72 h were determined via MALDI-MS. Transmission electron microscopy with X-ray microanalysis was used to detect putative co-localization of the cyclotides with Zn or Pb in the cells of *V. tricolor* treated with the highest concentration of heavy metals for 72 h. Cyclotide biosynthesis was dependent on the type of heavy metal and its concentration, time of treatment, plant species, and population type (MET *vs.* NMET). It was positively correlated with the level of tolerance of particular *Viola* species. The increased production of cyclotides was observed in the cells of metallophyte species, mostly in Zn-treated cells. The nonmetallophyte—*V. uliginosa* presented a decrease in the production of cyclotides independent of the dose and duration of the metal treatment. Cyclotides co-localized with Pb more evidently than with Zn, suggesting that cyclotides have heavy metal affinity. *V. lutea* ssp. *westfalica* transcriptome mining yielded 100 cyclotide sequences, 16 known and 84 novel named viwe 1–84. These findings support the hypothesis that cyclotides are involved in certain mechanisms of plant tolerance to heavy metals.

## Introduction

The Violaceae family, found worldwide, is rich in metallophyte species that colonize soils anthropogenically or naturally contaminated with heavy metals. In the *Viola* genus (violets), several types of metallophytes can be distinguished: obligatory metallophytes occurring exclusively on heavy metal-polluted soils (e.g., *V. lutea* ssp. *westfalica*), facultative metallophytes inhabiting both noncontaminated and contaminated soils (e.g., *V. tricolor*, metallicolous and nonmetallicolous populations), and accidental metallophytes occurring on polluted soils occasionally (e.g., *V. arvensis*). There are also nonmetallophytes with very narrow ecological requirements that do not occur on metalliferous soils (e.g., *V. uliginosa*) but show tolerance to heavy metals when they are applied to the cell suspension culture^1–3^. This suggests that violets developed so-called constitutive (innate) tolerance to heavy metals. Compared to the metallophyte species from other families (*Armeria maritima*, *Silene vulgaris* ssp. *humilis*, *Arabidopsis halleri*) among violets, even nonmetallophyte exhibited higher tolerance to heavy metals^3–5^.

The cyclotide family (cyclic, cysteine-rich peptides) represents a class of natural products with a wide range of biological and chemical properties, such as molecular stability and resistance to high temperatures and antifungal, antimicrobial, insecticidal, molluscicidal, cytotoxic or anti-HIV effects^6–12^. Although the biological roles of cyclotides in plants are not fully understood, it appears that their primary function is plant defense^7,9^. Similar to other peptides—defensins—most of the activities of cyclotides are associated with their ability to bind to phospholipids and disrupt biological membranes^13^. Cysteine residues in proteins are associated with various types of plant stress resistance^14^. Hypothetically, cyclotides or their precursors, which contain six cysteine residues, have the potential to bind to metal ions^15^ and could be involved in the response to abiotic stresses. This finding was supported by a study on *Viola baoshanensis*, a cadmium (Cd) hyperaccumulator*,* by Zhang et al.^16^. Cyclotides are produced by plants in very high amounts, up to 1.5 g in 1 kg wet mass^17^. Typically, a single plant often produces approximately 10–160 different cyclotides distributed in the cells of various tissues^18,19^. The different cyclotide sequences potentially have distinct biological activities and play specific roles^20^.

Cyclotides are found in species from several plant families (Cucurbitaceae, Fabaceae, Poaceae, Rubiaceae, Solanaceae, and Violaceae)^21–26^. Violaceae is a good model for studying the role of cyclotides because these peptides are biosynthesized by all species, unlike other cyclotide-producing families^24^.

Recent studies have shown that a quick cell reaction to applied heavy metals can be observed when the agent is added to the cell suspension culture^3,4^. Zn and Pb are deposited mainly in cell walls and vacuoles^3^. Cyclotides are stored mostly in vacuoles, where they can potentially interact and sequester heavy metals therein^27,28^.

The main goal of this study was to confirm the hypothesis that cyclotides could play a role in protecting plants against heavy metals by showing the following: (1) the level of cyclotide after metal (Zn, Pb) application to the cell suspension cultures of *Viola* species, representing different levels of tolerance to heavy metals; and (2) the colocalization of cyclotides with heavy metals (Zn, Pb) in plant cells.

## Materials and methods

### Plant material

The protocols for callus induction and initiation of cell suspensions from leaves of *V. lutea* ssp. *westfalica*, *V. tricolor* (NMET—nonmetallicolous and MET—metallicolous genotypes), *V. arvensis,* and *V. uliginosa* under in vitro conditions were developed and described by Sychta et al.^3^. All species were identified by prof. E. Kuta from the Jagiellonian University in Kraków. Since *V. uliginosa* and *V. lutea* ssp. *westfalica* are under legal protection in Poland and Germany, respectively, thus we used seeds of *V. uliginosa* collected by dr B. Slazak with the permissions of the Regional Directorate for Environmental Protection for previous studies^29^. Seeds of *V. lutea* ssp. *westfalica* were commercially bought from RarePlantsEU (www.rareplants.de). A deposited in KRA Herbarium of the Institute of Botany (Jagiellonian University in Kraków, Poland) voucher specimens represent three investigated species: *V. tricolor* MET (voucher no: KRA0369486), *V. arvensis* (voucher no: KRA0239632) and *V. uliginosa* (voucher no: KRA0247775) from PL-Bukowno, PL-Kraków and PL-Nowa Dęba. Experimental research, including the collection of plant material, complied with relevant institutional, national, and international guidelines and legislation.

The heavy metals Zn (Zn(NO_3_)_2_) and Pb (Pb(NO_3_)_2_) at concentrations of 200 µM and 2000 µM were applied during the exponential phase of suspension growth, which occurred on the 10th day after passage, as established previously by Sychta et al.^3^ and the cells were collected after 24 and 72 h of treatment. A suspension without heavy metals was used as a control and was collected on the 13th day after passage, corresponding to 72 h of treatment. All treatments were prepared in three biological repetitions.

### Preparation of extracts and MALDI-MS analysis

Fresh cells were centrifuged in a density gradient according to Sychta et al.^3^ to obtain viable cells, which were then immediately frozen in liquid nitrogen and freeze-dried (Freeze Dry System, Labconco). Each sample was placed in separate 2 ml Eppendorf tube and weighed. Then, the samples were powdered using a TissueLyser (Qiagen, Germantown, MD) for 1 min at 25 Hz and macerated for 2.5 min at 25 Hz in 30% acetonitrile (ACN) and 0.1% trifluoroacetic acid (TFA) in Milli-Q water (1:9, m/v). The tubes were centrifuged and the supernatant was collected.

The matrix-assisted laser desorption/ionization mass spectrometry (MALDI-MS) method developed and tested for cyclotides in several previous studies^30–32^. In short, the 4 × diluted extracts (0.5 µl of each sample) were spotted on a metal plate, air-dried, and sprayed with a 35 mg/mL in 50% ACN 2,5-dihydroxybenzoic acid, 0.2% TFA MALDI matrix solution using an automatic matrix sprayer (TM-Sprayer, HTX Technologies, Chapel Hill, NC). All the spots were analyzed using a MALDI Fourier transform ion cyclotron resonance (FTICR) (solariX 7 T-2ω, Bruker Daltonics, Bremen, Germany) mass spectrometer equipped with a Smartbeam II 2 kHz laser operated in positive ionization mode, externally calibrated using red phosphorus. Several measures were taken to ensure the best accuracy and reproducibility of the analysis. Firstly, the extract spots were scanned/imaged and averaged spectra were used. The images of the spots were taken in 250 µm lateral resolution which resulted in approx. 70 pixels per spot. The average mass spectra were generated in FlexImaging, version 4.0 (Bruker Daltonics), and the relative quantitative analysis (the mean intensity per pixel per spot) was performed in msIQuant software^33^. The same as in Slazak et al.^30^, the cyclotides produced by a particular species were identified by their monoisotopic molecular mass between 2.8 and 3.8 kDa. Serial dilutions of extracts from different species were also spotted on MALDI-MS target plates and analyzed in the same manner. Ions matching the following criteria were included in the analysis: monoisotopic ions not overlapping with others in the average spectra and showing a linear relation of abundance to signal intensity for any of the species in the analysis of extract dilutions. The monoisotopic molecular weights (MWs) of the analyzed cyclotides were compared against the masses of the cyclotides stored in Cybase (http://www.cybase.org.au/)^34^ for possible identification.

To validate of the MALDI-MS method, the LC-MS analysis was performed for two selected peptides (Suppl. 1).

### Co-localization of cyclotides and heavy metals—Immunogold and transmission electron microscopy (TEM) with X-ray microanalysis

Control cells of *V. tricolor* MET (known for the accumulation of metals in the cells structures) and cells treated with 2000 μM Zn or 2000 μM Pb for 72 h were fixed in 4% paraformaldehyde and 0.25% glutaraldehyde in phosphate buffer (PBS, 0.05 M, pH 6.8) for 90 min at room temperature (RT) and subsequently shaken over a period of time. Then, the fixed cells were rinsed in PBS for 15 min three times and dehydrated in an acetone series (10, 30, 50, 70, 90, 96, 100%). Dehydrated cells were embedded in LR White resin (Polysciences, USA) and sectioned with a glass knife using an ultramicrotome (Leica, Austria) into ultrathin sections collected on nickel grids coated with Formvar^3,35^. Then, the sections were blocked with 3% BSA (bovine serum albumin) in PBS for 1 h at RT. The immunostaining procedure was similar to that described by Slazak et al. (2018)^7^. The sections were incubated with the primary anti-cyclotide antibody (1:4000 dilution)^28^ for 24 h at 4 °C, rinsed four times in PBS, and incubated with the secondary antibody conjugated with 10 nm gold particles (anti-rabbit IgG [whole molecule] —gold antibody produced in goat, Sigma‒Aldrich, USA) at a dilution of 1:50 for 4 h at room temperature. The samples were examined via TEM with energy dispersive spectrometry (EDS) X-ray microanalysis using a JEM 1400 transmission electron microscope (JEOL Co., Japan) equipped with an 11-mpx MORADA G2 camera (EMSIS GmbH, Germany) and an X-ray microanalysis system (EDS INCA Energy TEM, Oxford Instruments, UK). The co-localization of Pb or Zn with cyclotides in the cells was determined by mapping the distribution of the elements (Pb or Zn and Au) in the cell compartments via X-ray mapping. All treatments were prepared in two biological and three analytical replicates.

### *V. lutea* ssp. *westfalica* transcriptome sequencing and mining of cyclotides

Transcriptome sequencing and analysis of the nontreated calli were performed according to Slazak et al.^30^. mRNA extraction, cDNA library preparation, and sequencing were outsourced to an outside provider, Macrogen, Inc. (Seoul, South Korea). *V. lutea* ssp. *westfalica* cell culture samples obtained according to the protocol described by Sychta et al.^3^ were studied for the presence of cyclotides for the first time. The sample was sent to Macrogen on ice and preserved in RNAprotect Tissue Reagent (Qiagen, Germany). Standard procedures were applied: Libraries were prepared using a TruSeq stranded mRNA kit (Illumina, San Diego, USA), followed by Illumina NovaSeq 2 × 100 bp paired-end 100 mln read sequencing. The resulting raw RNA-seq data were used to assemble the transcriptome de novo using Trinity^36^. The assembled transcriptome was queried using the NCBI-BLAST + service in the Ugene software package (v.1.31.0) for sequences similar to cyclotides stored in Cybase (http://www.cybase.org.au/) and using motif search (C-x(0,1)-[ES]-S-C-[AV]-[MFYW]-I-[PS]-x(0,1)-C) performed using Fuzzpro of EMBOSS (v. 5.0.0)^37,38^. The MWs of the identified cyclotides were calculated and compared with MALDI-MS experimental data. A sequence was considered novel if it had not been described before and was deposited in Cybase. The sequencing data was deposited in NCBI SRA, accession ID: PRJNA1111445.

### Statistical analysis

One-way ANOVA followed by Tukey’s post hoc test (p ≤ 0.05) was performed to determine the significance of differences in the relative abundances of the cyclotides between the control cells and the cells treated for 72 h with respect to the metal, dose, and selected species/genotype. ANOVA for repeated measures followed by Tukey’s post hoc test was performed (p ≤ 0.05), to determine the significance of differences in the relative abundances of cyclotides between cells treated for 24 and 72 h with different metals, doses, and selected species/genotypes. All analyses were performed with STATISTICA 13 and Microsoft Excel 365.

## Results

### Cyclotide biosynthesis is upregulated in the cells of metallophytes and downregulated in nonmetallophyte cells

**Figure 1:**
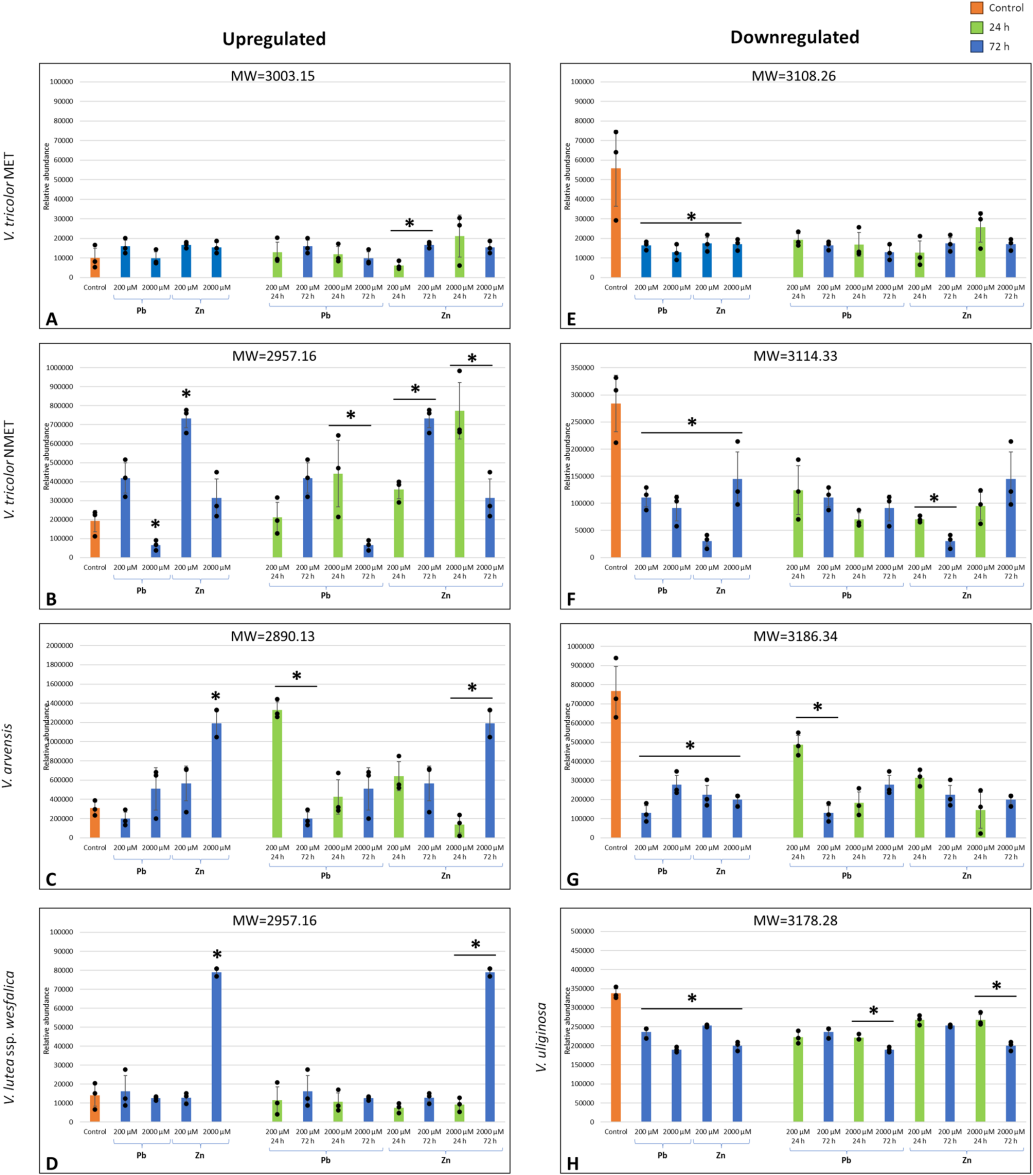
Mean relative quantities of selected—up- (**A**–**D**) and downregulated (**E**–**H**) cyclotides in the cells of metallophyte and nonmetallophyte violets, between control and 72 h of Zn or Pb treatments (left part of the graphs) and between 24 and 72 h of Zn or Pb treatments (right part of the graphs) in particular concentrations. Asterisks indicate statistical significance at p < 0.05 by one-way ANOVA followed by Tukey’s post hoc test (to determine the significance of differences in the relative abundances of the cyclotides between the control cells and the cells treated for 72 h with respect to the metal, dose, and selected species/genotype) or ANOVA for repeated measures followed by Tukey’s post hoc test (to determine the significance of differences in the relative abundances of cyclotides between cells treated for 24 and 72 h with different metals, doses, and selected species/genotypes). *V. tricolor* MET (**A**, **E**), *V. tricolor* NMET (**B**, **F**), *V. arvensis* (**C**, **G**), *V. lutea* ssp. *westfalica* (**D**), *V. uliginosa* (**H**). All treatments were prepared in three biological repetitions. For results of all analyzed cyclotides see Table 1 and Suppl. 2.

MALDI-MS analysis revealed peptide distribution patterns characteristic of cells of particular plant species. Fifty-five different cyclotides were identified, selected for analysis, and compared in extracts from the cell cultures of the selected *Viola* species. The effect of heavy metals on cyclotide biosynthesis was dependent on the metal concentration, treatment time, plant species, and origin of the plant material (MET *vs.* NMET). Compared with their respective nontreated controls, different cyclotides were up- or downregulated in the cells of specific plant species (Fig. 1, Table 1, Suppl. 2).

**Table 1:**
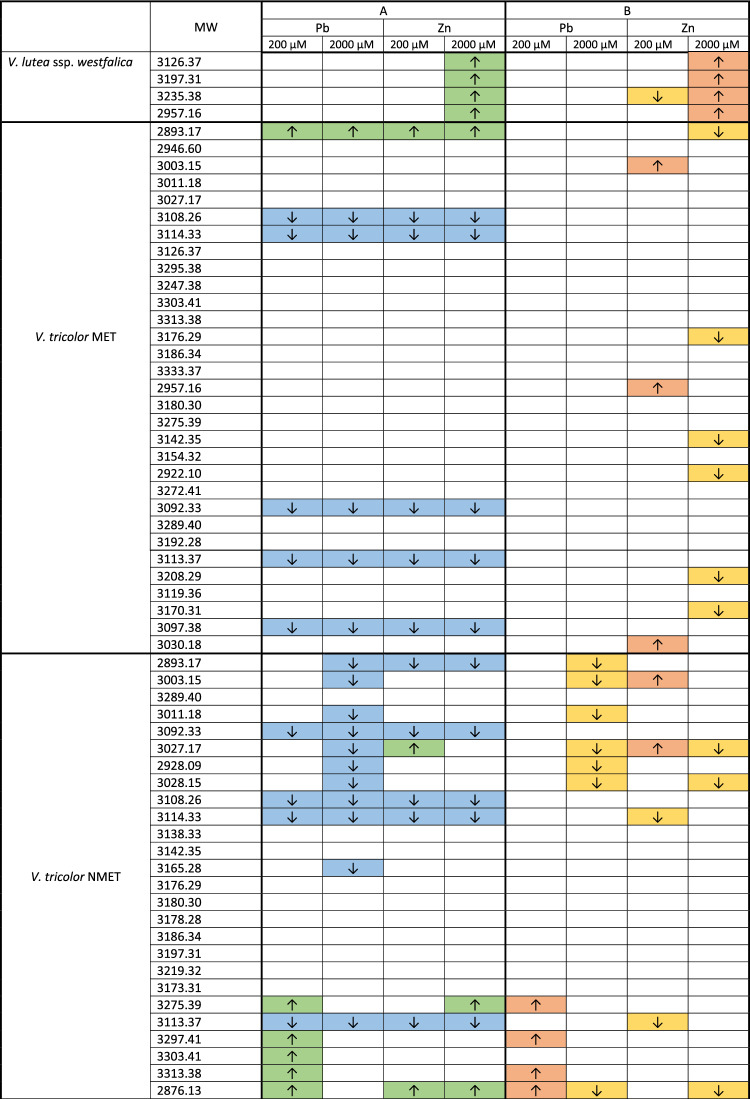

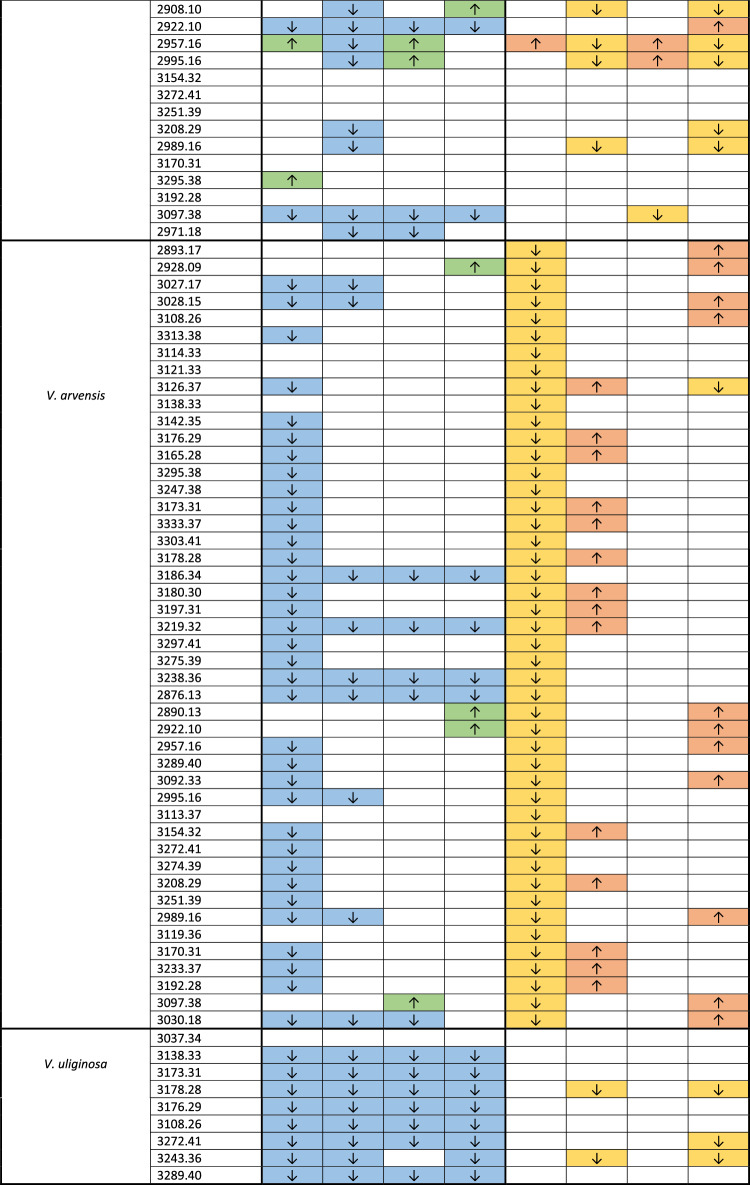
Mean relative quantities of cyclotides in particular species/genotypes of violets after treatment with Zn or Pb in concentrations of 200 µM and 2000 µM between control and 72 h of treatment (column A) and between 24 and 72 h of treatment (column B). Arrows up (green, orange) indicate a statistically significant increase and arrows down (blue, yellow) decrease of cyclotides. Colorless cells indicate lack of statistical changes in cyclotide content.

Among four cyclotides found in the obligate metallophyte *V. lutea* ssp. *westfalica*, all were upregulated after 72 h of treatment with 2000 µM Zn compared to those in the control cells and cells treated for 24 h. The decrease in abundance was visible only in the case of one cyclotide [MW = 3235.38] after 72 h of treatment with 200 µM Zn compared to that after 24 h of treatment but not in the comparision with the control. Treatment with Pb did not influence cyclotide production in this species (Table 1, Suppl. 2).

In *V. tricolor* MET, among 31 analyzed cyclotides, three were upregulated during treatment with low doses of Zn (200 µM) compared with 24 and 72 h of treatment and one was upregulated after 72 h of treatments with Pb and Zn in all concentrations compared to this in the control cells. Five cyclotides were downregulated after 72 h compared to those in the control cells (in all treatments) and 6 compared to those treated for 24 h (in treatment with 2000 µM Zn) (Table 1, Suppl. 2).

In *V. tricolor* NMET, among 40 analyzed cyclotides, 4 were upregulated after 72 h of treatment with 200 µM Pb, 3 were upregulated after 72 h of treatment with 200 µM Zn and 3 were upregulated after 72 h of treatment with both metals compared to those in the control cells. Compared with those in the control cells, 8 cyclotides were downregulated after treatment with both metals, and 11 after treatment with 2000 µM Pb. Comparing cyclotides production after 24 h to 72 h of treatment with heavy metals, 4 cyclotides were upregulated in the cells treated with 200 µM Pb, 3 cyclotides were upregulated in the cells treated with 200 µM and one in the cells treated with 2000 µM Zn. One cyclotide was upregulated in cells treated with low doses (200 µM) of both metals. Altogether, 7 cyclotides were downregulated at high concentrations (2000 µM) of Pb or Zn, 5 were downregulated after treatment with 2000 µM Pb and 3 after treatment with 200 µM Zn (Table 1, Suppl. 2).

In *V. arvensis,* an accidental metallophyte, among 46 analyzed cyclotides, 4 were upregulated after Zn treatments (3 after low and one after high doses) and 35 were downregulated (30 in the Pb treatment group and 5 in the treatments with both metals) compared to those in the control group. Treatment with 200 µM Pb decreased the biosynthesis of all cyclotides (except one cyclotide which was also decreased after treatment with 2000 µM Zn) compared 24 h to 72 h of treatment, whereas 14 cyclotides were upregulated after treatment with 2000 µM Pb and 11 after treatment with 2000 µM Zn (Table 1, Suppl. 2).

In the nonmetallophyte *V. uliginosa,* among nine analyzed cyclotides, 8 were downregulated after almost all the treatments compared with 72 h of treatment and the control group. There was no significant difference in the cyclotide content (MW = 3037.34) between the control cells and the cells treated with heavy metals for 72 h. However, changes in the cyclotide content in the cells between 24 and 72 h of treatment were detected only in 3 of the cyclotides treated with 2000 µM Pb and Zn (Table 1, Suppl. 2).

### A large variety of novel cyclotides is found in the rare obligate metallophyte *V. lutea* ssp. *westfalica*

The transcriptomes of *V. lutea* ssp. *westfalica* callus tissue were mined for cyclotide sequences. This approach yielded a host of 100 sequences, 16 of which were previously described from other plant species and 84 of which were novel (Table 2). The novel cyclotides were named viwe 1–84 and ordered according to their increasing molecular weights. Two of the peptides (varv peptide F and vive 48) were among the cyclotides detected in the MALDI-MS quantitative analysis.

**Table 2:** The basic characteristics of cyclotides found in *V. lutea* ssp. *westfalica* callus tissue transcriptome—amino acid sequence and calculated molecular weights (MW).

Name	Sequence	MW
Viul D	GIPCGESCVWIPCLTSAIGCSCKSKVCYKN	3124.32
viul M	GIPCGESCVFIPCLTAAIGCSCKSKVCYRN	3097.32
vitri A	GIPCGESCVWIPCITSAIGCSCKSKVCYRN	3152.33
vitri 98	GIPCGETCVFSGCYSVTFGCACEKRVCYKNu	3199.26
Part of vitri 60 precursor	GIPCGETCIFGRCHTGIIGCACEKYMCCKN	3185.24
vitri 42a	GGTIFNCGESCFQGTCYTKGCACGDWKLCYGEN	3433.25
vitri 28 (incomplete)*	VPSSDCLETCFGGKCNAHRCTCSQWPLCAKN	3333.32
vinc A (incomplete)*	PVCGETCTLGTCYTAGCSCSWPVCTRN	2787.01
varv peptide F	GVPICGETCTLGTCYTAGCSCSWPVCTRN	2957.12
varv peptide D	GLPICGETCVGGSCNTPGCSCSWPVCTRN	2876.07
Kalata S	GLPVCGETCVGGTCNTPGCSCSWPVCTRN	2876.07
Kalata B1 (incomplete)*	LPVCGETCVGGTCNTPGCTCSWPVCTRN	2833.07
CyO8	GTLPCGESCVWIPCISSVVGCSCKSKVCYKN	3225.37
CyO4	GIPCGESCVWIPCISSAIGCSCKNKVCYRN	3165.33
CyO3	GIPCGESCVWIPCLTSAIGCSCKSKVCYRN	3152.33
CyO12	GLPICGETCVGGTCNTPGCSCSWPVCTRN	2890.09
viwe 1	GLPVCGETCVGGPCNTPGCSCSRPVCTRN	2842.10
viwe 2	GLPICGETCVGGTCNTPGCICSWPVCTTN	2861.09
viwe 3	GIPICGETCVGGTCNTPGCSCSWPVCVRN	2888.11
viwe 4	GIPICGETCVGGTCNTPGCSCSWPVCTRN	2890.09
viwe 5	GLPVCGETCVGGTCNTLGCSCSWPVCTRN	2892.11
viwe 6	GMPVCGETCVGGTCNTPGCSCSWPVCTRN	2894.03
viwe 7	GLPVCGETCVGGTCNTPGCSCSWPVCKRN	2903.12
viwe 8	GLPICGETCVGGSCNTPGCSCSWPMCVRN	2906.07
viwe 9	GLPVCGETCVGGTCNTPGCSCSWPVCMRN	2906.07
viwe 10	GLPICGETCVGGTCNTPGCSCSWPVCMRN	2920.08
viwe 11	GIPICGETCTLGTCYTAGCSCSWPVCTRN	2971.14
viwe 12	GLPICGETCTLGTCYTAGCSCSWPVCTRN	2971.14
viwe 13	GVPICGETCTLGTCYTAGCTCSWPVCTRN	2971.14
viwe 14	GHCGESCMVLPCFTASRGCSCSGAICWKN	2982.11
viwe 15	GVPICGDTCFGGTCYTPGCSCSWPVCMRN	2989.07
viwe 16	SIPCGESCVFIPCLTGAIGCACKSSVCYLN	3013.24
viwe 17	GSAVPCGESCFFGGGCDTPGCSCTWPACTKN	3017.05
viwe 18	SIPCAESCAFVPCYGIIPCSCKNGICYSN	3017.18
viwe 19	GLPVCGETCLGGTCNTPGCSCSWPVCVKYD	3024.15
viwe 20	GSIFNCGETCILGTCYTPGCSCVYGACSKN	3026.13
viwe 21	GSIFNCGESCVLGTCYTSGCSCVYGLCSKN	3030.13
viwe 22	GSIFNCGESCVLGTCYTPGCSCVYGLCSKN	3040.15
viwe 23	GVACPETCIFTSCFITSCTCDHGRCRRN	3055.19
viwe 24	GIPCGESCVFIPCLTAAIGCSCSSKVCYRN	3056.26
viwe 25	GYPCVETCVFSGCFITNCICNYGSCVWN	3057.12
viwe 26	GSVFNCGESCLGGKCNTPDCTCSFPLCTKN	3060.15
viwe 27	GSVFNCGESCLGGTCNTPGCTCSSFPLCTKN	3062.13
viwe 28	GIPCAETCAFIPCMATAVFGCSCSNNVCYN	3064.13
viwe 29	GSIPCAESCAFVPCYGIIPCSCKNGICYSN	3074.20
viwe 30	GWSCQETCIFSSCYLTGCTCSYSVCKKN	3076.15
viwe 31	GIPCGESCVWIPCITAAIGCSCSSNVCYRN	3081.22
viwe 32	GIPCGESCVFIPCITAAIGCSCSNKVCYRN	3083.27
viwe 33	GIPCGESCVFIPCLTAAIGCSCSNKVCYRN	3083.27
viwe 34	GSVFNCGETCIWGTCYTPGCSCVYGACSKN	3085.11
viwe 35	GIPCGESCVCITCISSAIGCSCKIKVCYRN	3085.28
viwe 36	GIPCAESCVWIPCTITALLGCGCSNKVCYN	3093.28
viwe 37	GVPTCDESCVLGTCFTPDCTCSWPICLRN	3095.19
viwe 38	GIPCGESCVWIPCISSVIGCSCSSKVCYKN	3097.28
viwe 39	GQFCGESCIVSSCYITRCTCTANFCYRN	3104.16
viwe 40	GIPCGESCVWIPCISSVVGCSCSSKVCYRN	3111.27
viwe 41	GLYVCGERCLRGRCNAPGCICTNKICTKN	3120.36
viwe 42	GRFCLETCVFSSCFITGCDCEFTSCFKN	3122.17
viwe 43	GIPCGESCVWIPCITAAIGCSCSNKVCYRN	3122.28
viwe 44	GIPCGESCVWIPCVTVVIGCSCSSNVCYRN	3123.27
viwe 45	GIPCAESCVWIPCTITALLGCSCSNKVCYN	3123.29
viwe 46	GSIFKCGETCVLGTCYTPGCHCIWGVCAKN	3125.26
viwe 47	GVLPCGESCVFIPCVTAVIGCACKSSVCYKN	3125.34
viwe 48	GSSCYESCYLIPCITSIAGCSCNQNTCTDD	3126.09
viwe 49	GDVCTETCFTDYCFLGGCTCYWPVCKKN	3131.16
viwe 50	GPLCGDTCVYDPCLISAPCKCKNKVCYRN	3138.31
viwe 51	GSIFNCGETCLLGTCYTSGCSCVYRVCSKD	3144.20
viwe 52	GIPCGETCLFGGCNTSIFGCACEKRVCYKN	3148.26
viwe 53	GSIPCGESCVWIPCISGIAGCSCSNKVCYKN	3153.28
viwe 54	GIPCGITCELNPCHSFVPCTCQHRVCYSN	3156.24
viwe 55	GVPCGESCVFNPCLTGVVLCRCSSYVCFKD	3160.29
viwe 56	GSVPCGESCVWIPCISAVVGCSCSNKVCYKN	3167.29
viwe 57	GSTPCGESCVWIPCISAVVGCSCSNKVCYKN	3169.27
viwe 58	GSTPCGESCVWIPCISSVVGCSCSNKVCYLN	3170.26
viwe 59	GSTPCGESCVWIPCISAVVGCSCSNKVCYMN	3172.22
viwe 60	GRVPCGETCSLGTCYFAGCTCDWPICWRN	3173.20
viwe 61	GSTPCGESCVWIPCISSIVGCSCSSKVCYMN	3175.22
viwe 62	GLTCVESCIVIPCVTGLLGCYCSNHICYKN	3180.35
viwe 63	GSIPCGESCVWIPCISAVVGCSCSNKVCYKN	3181.31
viwe 64	GIPCGESCVWIPCLTSTIGCSCKSKVCYRN	3182.34
viwe 65	GVGAYCGESCVLIPCLSAIIGCSCSNSDCFKN	3188.27
viwe 66	SIPCAESCVWIPCISSVVGCSCKSKVCYRN	3196.36
viwe 67	GIPCGETCIFSGCYSVTFGCACEKRVCYKN	3213.28
viwe 68	GVLPCGESCVWIPCISSVVGCSCKSKVCYKN	3223.39
viwe 69	GFSCMESCILLPCATKYIGCSCENKLCVKN	3232.35
viwe 70	GSSKCWETCVLIPCATAILGCSCKDFICVKN	3267.42
viwe 71	GIPCGESCVWIQCISSAIGCYCNNKVCFWN	3272.29
viwe 72	GLVSCGQSCYRTPCVALTTCSCRFHVCYKN	3274.35
viwe 73	GTIFDCGESCLLGKCYTPGCACGSWGLCYGQN	3281.23
viwe 74	GTIFDCGESCVFGTCYTPGCACGSWALCYGQN	3288.17
viwe 75	GTFPCGESCVWIPCLSKVIGCACKSKVCYKN	3298.44
viwe 76	GLNCGETCWGFSCDRDDCSCWTWPYCSKN	3311.12
viwe 77	GTIFDCGESCLLGTCYTKGCSCGSWKLCYGTN	3345.28
viwe 78	GWLHCGETCRITPCLAADLMCFCTDGVCLRN	3376.37
viwe 79	GSQHCAETCYLIPCLTTQIGCSCINRACYRN	3396.39
viwe 80	SLFNCGETCLWGTCYTPGCNCNKWRVCEKN	3404.32
viwe 81	GWISCVEACYYLPCASRVFGCSCVRNVCMRN	3464.41
viwe 82	GTIFNCGESCFHGKCYTKGCACGDWKLCYGEN	3469.30
viwe 83	GEHCYEVCYFNPCVTRLLGCYCHLHMCIKN	3523.41
viwe 84	GQARFCHETCTLNPRCITAQFGCYCTHRVCTIN	3721.55

### Cyclotides co-localize with Zn and Pb in *V. tricolor* cells imaged via TEM

**Figure 2:**
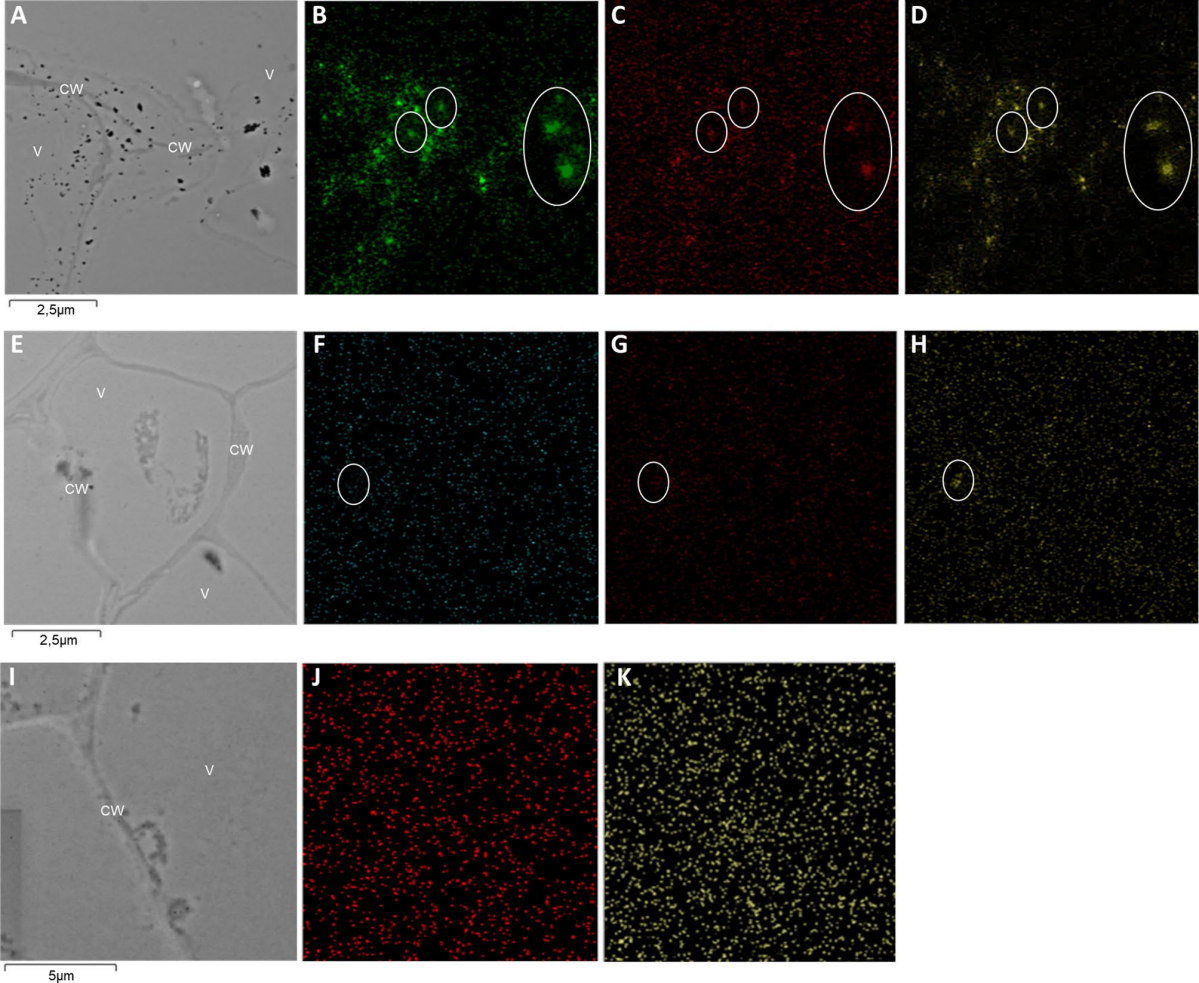
TEM microphotographs of *V. tricolor* cells from suspensions treated with 2000 µM of Pb (A–D) or Zn (E–H) for 72 h and control cells (I–K). The distribution maps show cell walls (CW) and vacuoles (V) treated with Pb (**A**) and Zn (**E**) that co-localize with lead (**B**, green points) or Zn (**F**, light blue points) deposits, gold particles (representing cyclotides) (**C**, **G** and **J**, red points) and sulfur deposits (**D**, **H** and **K**, yellow points). The circles show examples of co-localization sites for metals, sulfur and cyclotides. All treatments were prepared in two biological and three analytical replicates.

Using TEM, Pb and Zn were found to be deposited mainly in cell walls but also in vacuoles. On the microphotographs stained with the immunogold technique, Pb deposits co-localized with gold signals representing cyclotides (Fig. 2A–C). Gold signals were also co-localized with high amounts of sulfur, which is an important element of the cyclotide structure (cysteine side chain) (Fig. 2C,D). The Zn deposits were less visible than the Pb deposits and poorly co-localized with the Au signals and sulfur (Fig. 2E–H). In cells not treated with heavy metals (control cells) the Au and S signals are scattered (Fig. 2I–K).

## Discussion

The present study showed that the biosynthesis of some cyclotides changed significantly in cells treated with heavy metals compared with that in nontreated controls. This suggests that cyclotides may be involved in the mechanisms of tolerance to heavy metals. Generally, heavy metal-tolerant species develop various mechanisms to mitigate the effects of heavy metals, e.g., transmembrane transporters, cellular receptors, the production of different polymers (e.g., pectin, lignin, cellulose, hemicellulose), glutathione, nicotinamide, flavonoids, phenols, antioxidants, phytochelatins, metallothioneins, organic and inorganic acids, and the deposition of metals in cell walls or vacuoles^39–41^. Polyamines, as secondary metabolites, possess antioxidant properties, can bind heavy metals and have an influence on metal uptake by plants^42^. Among the heavy metal binding peptides, metallothioneins and phytochelatins are the best known compounds that play a major role in heavy metal detoxification and accumulation in tolerant plants^43^. Phytochelatins and metallothioneins interact with heavy metals via the thiol (-SH) group of cysteine to produce protein-metal complexes^44,45^. Additionally, the role of cyclotides in heavy metal tolerance is supported by the work of Zhang et al.^16^*.* In this study, a transformed yeast strain YL36 expressing cyclotides showed greater tolerance to Cu than strains not producing these peptides^16^. Cyclotide genes have also been shown to be upregulated in the Cd hyperaccumulating plant *V. baoshanensis* under Cd exposure, indicating that cyclotides may play a role in the response to heavy metals^16^.

The results of the current study show that the biosynthesis of some cyclotides in cells treated with heavy metals is significantly greater than that in control cells but mostly in cells of the metallicolous genotypes. Plants develop both constitutive (present in most genotypes including also nonmetallophyte species of a given genus) and adaptive (hypertolerance, present only in genotypes inhabiting polluted areas) mechanisms for coping with high heavy metal concentrations^46,47^. Our previous studies showed that the tolerance of violets to heavy metals is species- and population-dependent^3,48^. All analyzed *Viola* species developed constitutive tolerance to Zn and Pb, but metallophytes (obligate, facultative, accidental) exhibit hypertolerance to heavy metals^3^. The population of *V. tricolor* from contaminated soils is characterized by enhanced antioxidant machinery that provides protection from heavy metal toxicity^49^. It was also shown that the ZIP, HMA4 or Nramp families of heavy metal transporters (root-to-shoot transport) and the MTP3, COPT5, and ABC tonoplast transporters, which are all responsible for vacuole sequestration, play crucial roles in the tolerance of violets^50^. In contrast, the current study showed a decrease in the biosynthesis of almost all analyzed cyclotides after heavy metal treatments in the cells of the nonmetallophyte *V. uliginosa.* This further supports the hypothesis that cyclotide biosynthesis is related to the level of tolerance. It seems that the changes in cyclotide production in response to heavy metal stress may belong to the mechanisms underlying the hypertolerance of metallophyte violets.

In *V. tricolor* (both the MET and NMET populations) and *V. arvensis* cells, treatment with Pb decreased the biosynthesis of most of the cyclotides. In contrast, Zn treatments, especially at low doses in *V. tricolor* (MET and NMET) and at high doses in *V. arvensis*, upregulated cyclotide production between 24 and 72 h of treatment. This could be a result of the greater toxicity of Pb than Zn, which was also observed in the earlier study in suspension cultures treated with these metals at different concentrations^3^. Such an effect is likely because Pb, as a nonessential element, is generally more toxic than Zn^51^. This is manifested by a stronger initiation of programmed cell death by Pb than by Zn in *V. tricolor* cell suspension culture, as shown earlier^4^. In contrast, Zn is an essential metal that can support plant metabolism at relatively low concentrations, especially in the case of resistant species^52,53^, and can be responsible for promoting the cyclotide production observed in the present study. Surprisingly, in the accidental metallophyte *V. arvensis,* some cyclotides were upregulated between 24 and 72 h of treatment with high doses of Pb. However, the relatively high tolerance of this species to Pb was described in earlier studies^3^. The opposite hypothesis of decrease cyclotide content after Pb treatment is that some cyclotides are not visible in MALDI-MS analysis in cells treated with Pb (resulting in a change in molecular weight—not detectable in the selected spectral range). This may result from Pb binding to specific cyclotides what is visible in TEM micrographs with immunogold technique (Fig. 2B,C).

In the present study, the production of some cyclotides increased, whereas that of others decreased under treatment with heavy metals. This result is not surprising and reflects the process of adjusting the whole cyclotide production pattern to mitigate certain stresses. In an earlier study, we showed a similar type of response to biotic stress—spider mite infestation^32^. The production of individual cyclotides can be regulated at the gene expression level^19^. However, we also showed that cyclotide production patterns can be shaped by turnover processes. In this case, it would appear that the unnecessary cyclotides can be broken down, and the released resources can be reused^54^. Multiple studies have shown that cyclotide production patterns differ between habitats and seem to be shaped by the needs of a particular environment (e.g.,^30,55,56^).

Mining of the transcriptome of *V. lutea* ssp. *westfalica* cell cultures performed in the present study led to the discovery of 84 new and 16 known cyclotide sequences. A screening of plants from the Violaceae by Burman et al.^24^ revealed that most members of this family likely produce cyclotides. In this work, the authors estimated the number of different cyclotide sequences in the Violaceae to be dozens of thousands^24^. It seems that if such a large set of new sequences can be described from a single species of violet, as shown currently, these estimates are correct. Only four of the described sequences were present in the set of peptides analyzed quantitatively with MALDI-MS. The first reason may be the frequently noted mismatch of observed sequences between the peptide and the transcriptome levels, e.g., in Slazak et al.^30^. This may be caused by cyclotide stability when a peptide is no longer expressed and is not present in the transcriptome but is still detectable by MS. On the other hand, the expression levels may be low, or for some reason, the final cyclotide is not produced, leading to difficulties in detecting it in MS spectra. Finally, the chosen methodology for quantitative analysis and the criteria for the selection of peaks might have contributed to the low number of analyzed peptides.

The current study showed that heavy metals co-localize with cyclotides in the vacuoles of cells treated with heavy metals. It can be hypothesized that this is due to interactions between cyclotides and metals; it may be that the peptides can bind metals thus act as “chelators” and deposit them into vacuoles becoming non-toxic to other cell organelles^57^. It was previously described that Zn and Pb, as well as cyclotides, are deposited in vacuoles of *Viola* cells^3,28^. This co-localization supports other findings about the metal-binding properties of cyclotides, especially those of Mn ions^58,59^. Transcriptomic studies on *V. baoshanensis* also support this by showing that cyclotides contain zinc finger (ZF) proteins that can bind divalent metals^60^. It is known from ex vivo studies that divalent cation coordination is the invariant property of all cyclotides and that the Asn 23, Thr 24, and Glu 15 residues are involved in Mn-binding in Kalata B1^58,61^.

## Conclusion

The present study describes the involvement of cyclotides in tolerance to heavy metals in the cells of different *Viola* species. It was shown that cyclotide production patterns change significantly in response to Pb and Zn treatments and that the metals are sequestered in deposits in the vacuole, together with cyclotides. A number of new cyclotides possibly involved in the response to heavy metals were described from a rare obligate metallophyte, *V. lutea* ssp. *westfalica*.

### Supplementary Information

Supplementary Information 1.

Supplementary Material 1.

Supplementary Information 2.

Supplementary Material 2.

## Data Availability

All the data are available in the main text or supplementary materials. The datasets used and/or analyzed during the current study are available from the corresponding author on reasonable request. Herbarium specimens of investigated species are available in KRA Herbarium (Kraków, Poland): voucher no: KRA0369486 (*V. tricolor* NMET), voucher no: KRA0239632 (*V. arvensis*) and voucher no: KRA0247775 (*V. uliginosa*). The sequencing data from *V. lutea* ssp. *westfalica* is available in NCBI SRA, accession ID: PRJNA1111445.
