# Supplementary material for: The involvement of cyclotides in the heavy metal tolerance of Viola spp
Source: Sci Rep. 2024 Aug 20;14:19306. doi: 10.1038/s41598-024-69018-x (PMC11336087; doi:10.1038/s41598-024-69018-x)
Supplement: Supplementary file 1 — Supplementary Information 1. [file 41598_2024_69018_MOESM1_ESM.docx]

**Supplementary material 1.** Additional validation of MALDI-MS methods with LC-MS.
