# Supplementary material for: The involvement of cyclotides in the heavy metal tolerance of Viola spp
Source: Sci Rep. 2024 Aug 20;14:19306. doi: 10.1038/s41598-024-69018-x (PMC11336087; doi:10.1038/s41598-024-69018-x)
Supplement: Supplementary file 2 — Supplementary Material 1. [file 41598_2024_69018_MOESM2_ESM.pdf]

Supplementary material 1. Additional validation of MALDI-MS methods with LC-MS.

1. Material and Methods

All LC-MS analyses were performed using a UPLC Acquity Premier, Waters or nanoAcquity, Waters equipped in Kinetex 1.7 µm C18 100 Å LC column 100 x 2.1 mm, combined with MS QTof Xevo, Waters. All samples were separated in 5%-95% ACN, 0.1% FA gradient. The relative abundance of cyclotides MW = 3114.33 Da and 2893.17 Da was established in samples from *V. tricolor* NMET population control cell suspensions and ones treated with 2000 µM of Pb for 72 h. The m/z values corresponding to the monoisotopic 3+ peaks for a cyclotide were used to isolate the chromatographic peaks using the mass filtering tool. Subsequently, the peaks were integrated and the area under the curve (AUC) conveyed measures of relative abundance. One-way ANOVA was performed, followed by Tukey's test ($p \leq 0.05$) to determine the significance of differences in the cyclotides' relative abundances between the control and treated samples.

2. Results

The results of LC-MS experiments for selected cyclotides confirm the trends indicated using MALDI-MS (Fig. 1),

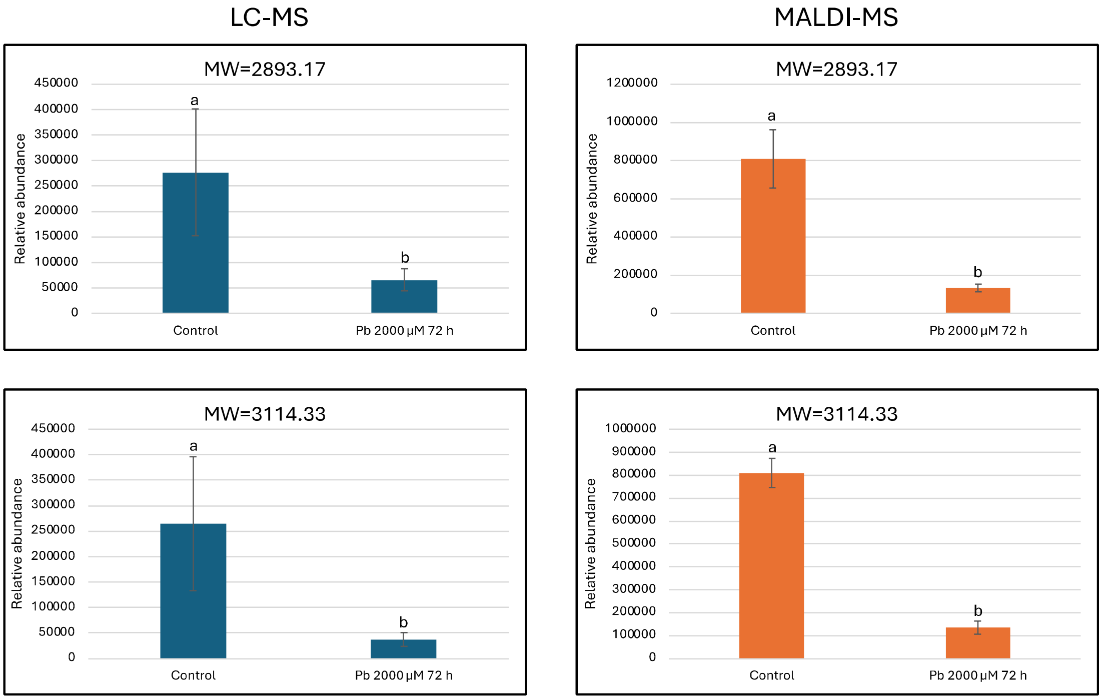

Figure 1. The comparison of mean relative quantities of selected cyclotides (3114.33, 2893.17) in *V. tricolor* NMET population control cell suspensions and ones treated with 2000 µM of Pb for 72 h, analyzed by LC-MS and MALDI-MS
