# Supplementary material for: The involvement of cyclotides in the heavy metal tolerance of Viola spp
Source: Sci Rep. 2024 Aug 20;14:19306. doi: 10.1038/s41598-024-69018-x (PMC11336087; doi:10.1038/s41598-024-69018-x)
Supplement: Supplementary file 3 — Supplementary Information 2. [file 41598_2024_69018_MOESM3_ESM.docx]

**Supplementary material 2.** Mean relative quantities of cyclotides in cells of *V. lutea* ssp. *westfalica*, *V. tricolor* MET, *V. tricolor* NMET, *V. arvensis* and *V. uliginosa*, between control cells and cells treated with Zn or Pb for 72 h (left part of the graph) and between 24 h and 72 h of treatments in particular concentrations (right part of the graph). Asterisks indicate statistical significance at p < 0.05 by one-way ANOVA or ANOVA for repeated measures.
