# Supplementary figures and images for: The involvement of cyclotides in the heavy metal tolerance of Viola spp
Source: Sci Rep. 2024 Aug 20;14:19306. doi: 10.1038/s41598-024-69018-x (PMC11336087; doi:10.1038/s41598-024-69018-x)

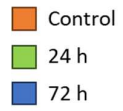

### *V. lutea ssp. westfalica:*

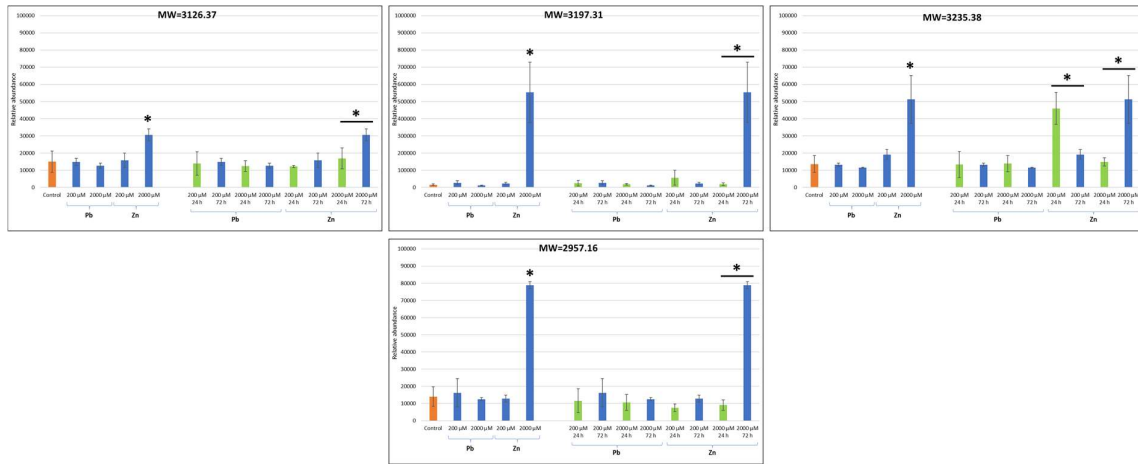

### *V. tricolor MET:*

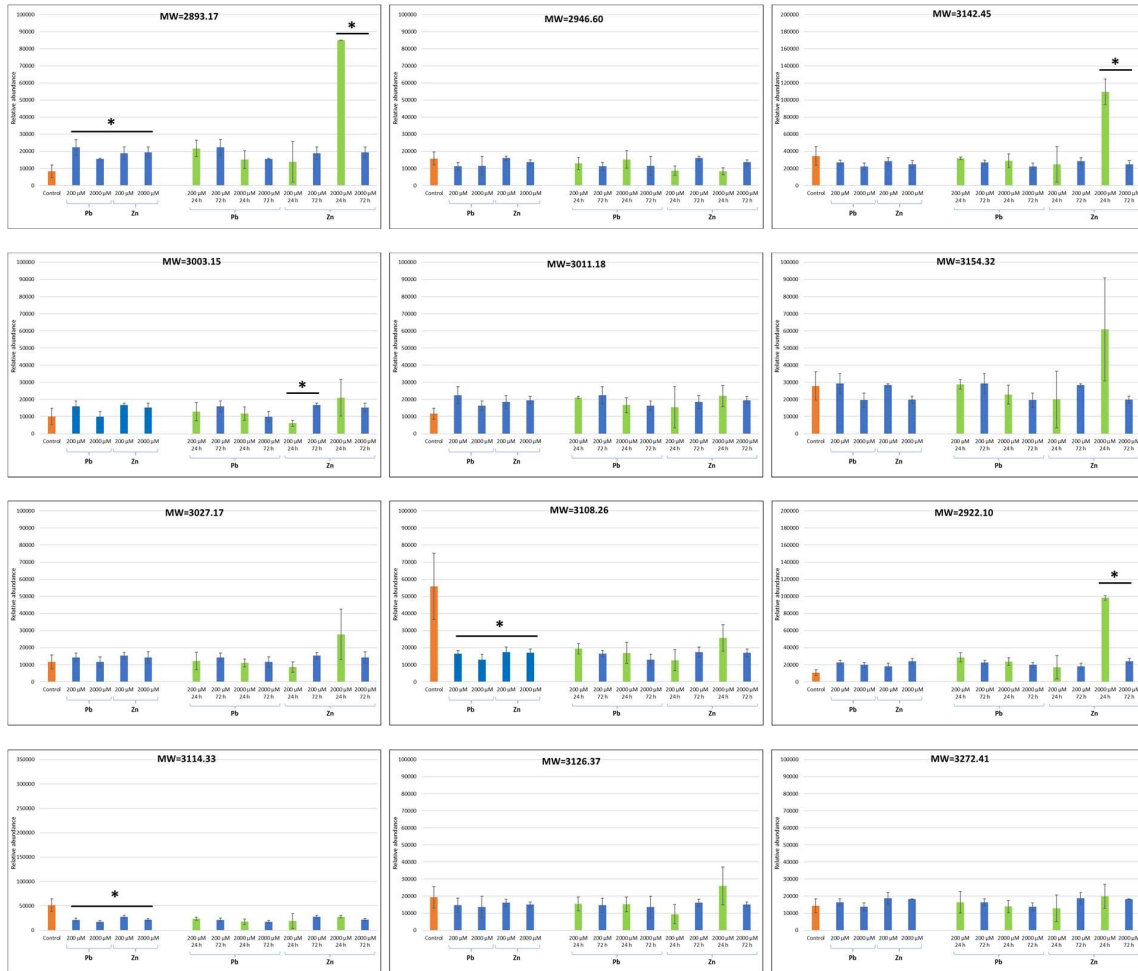

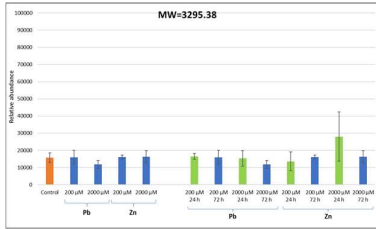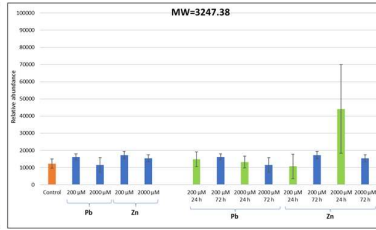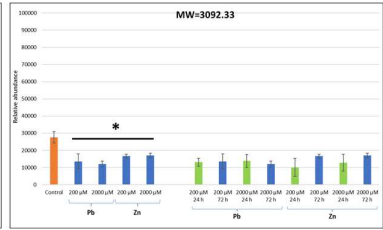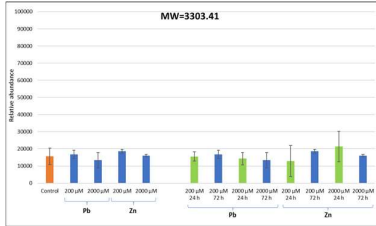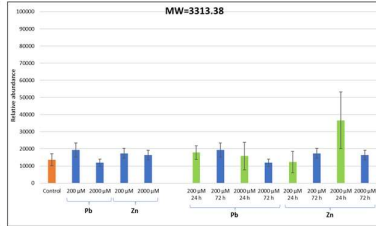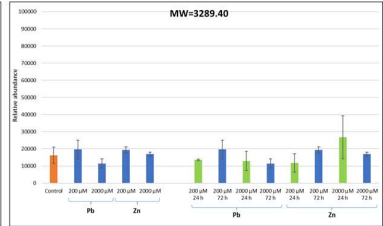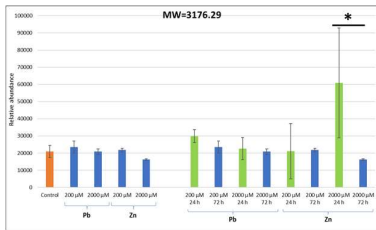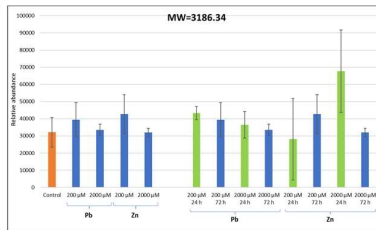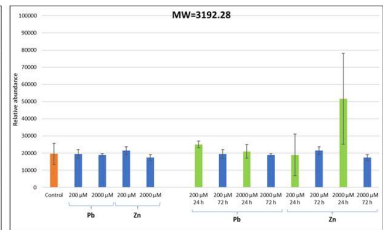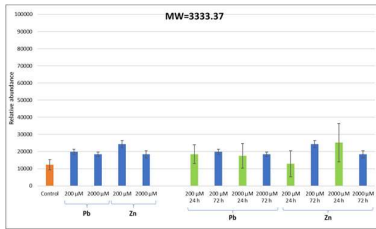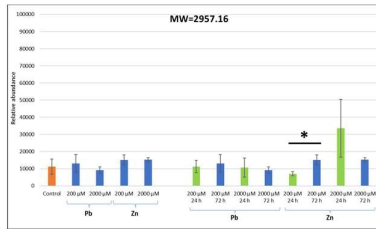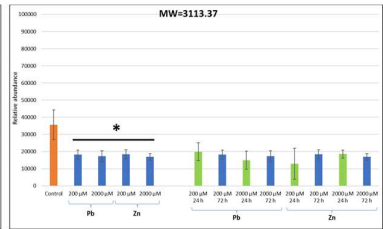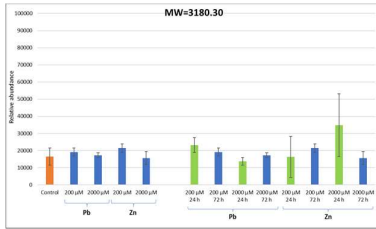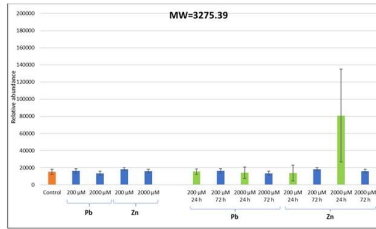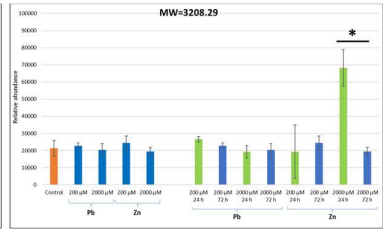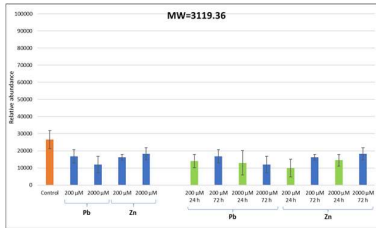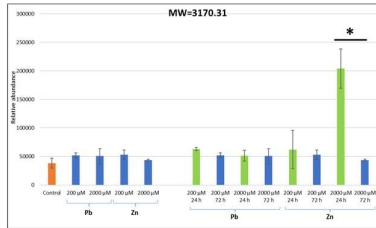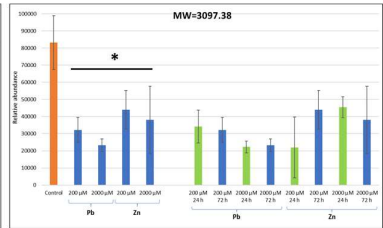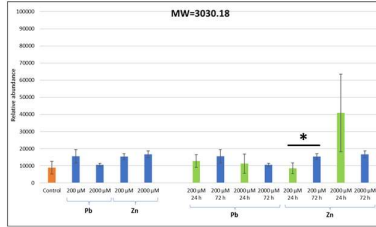

*V. tricolor* NMET:

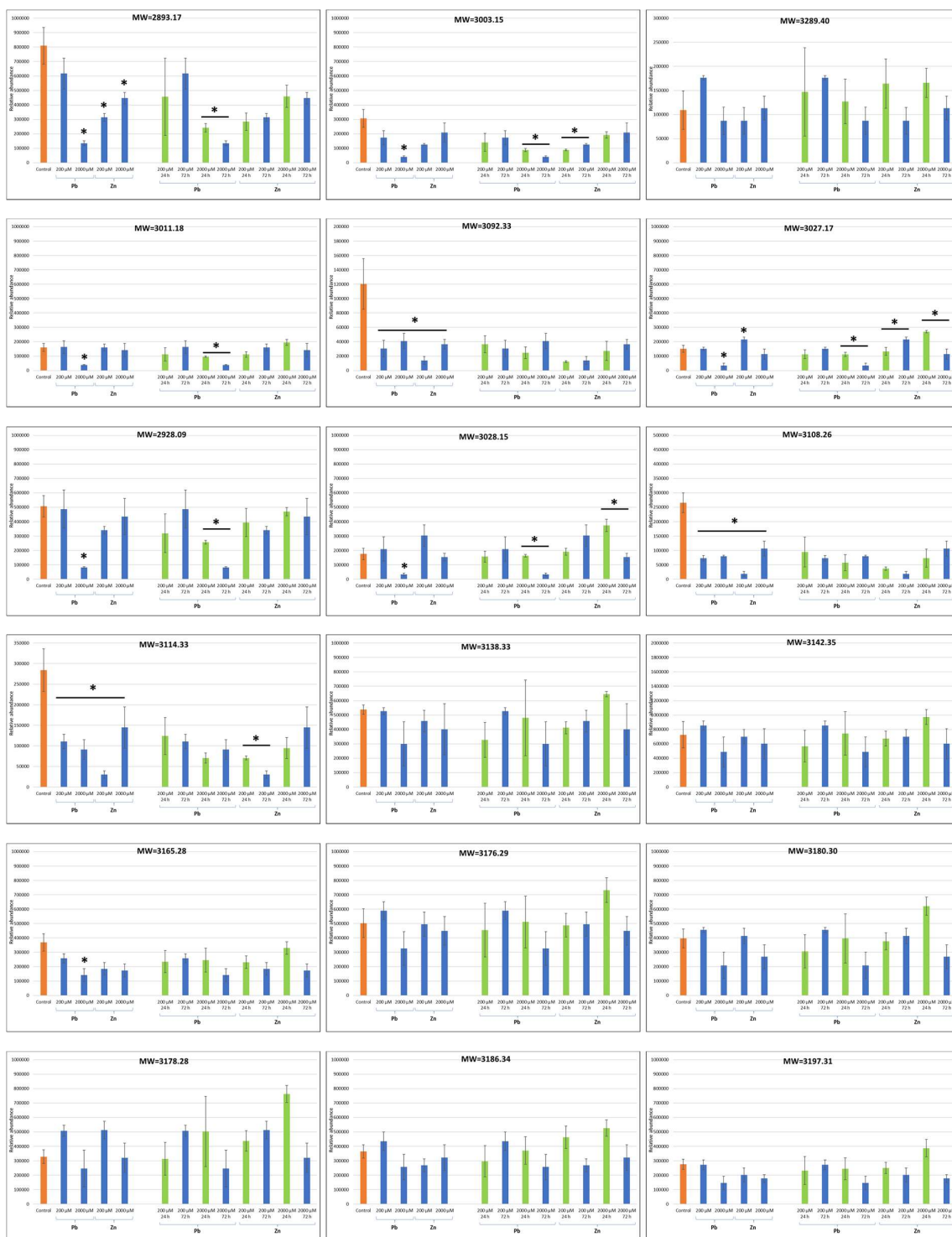

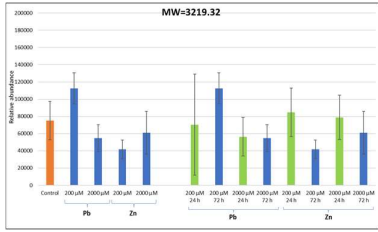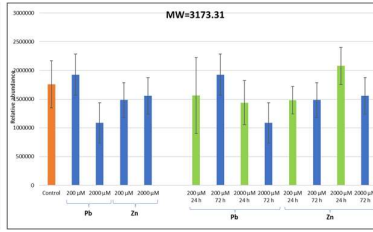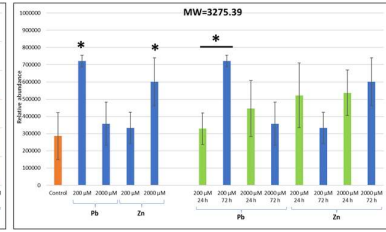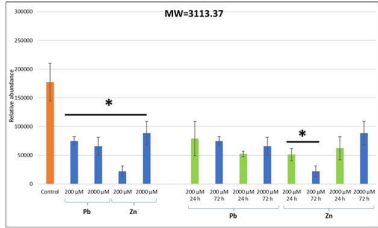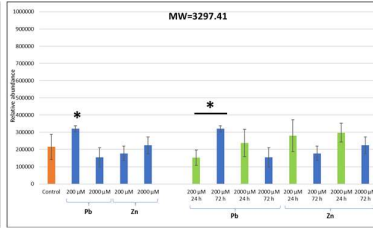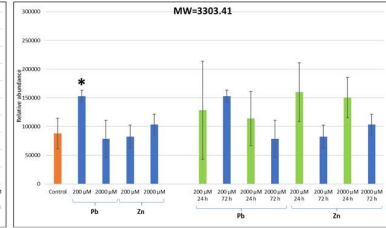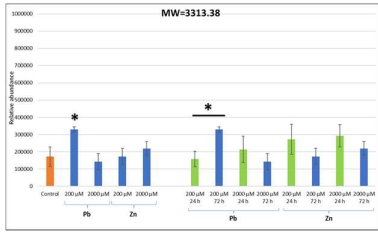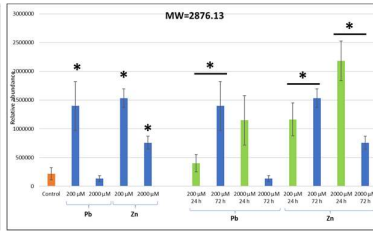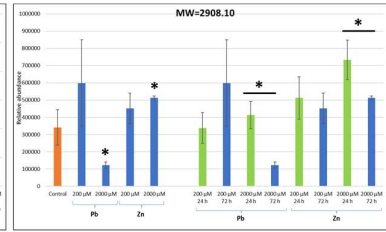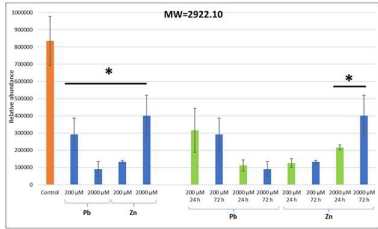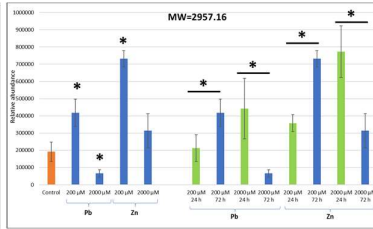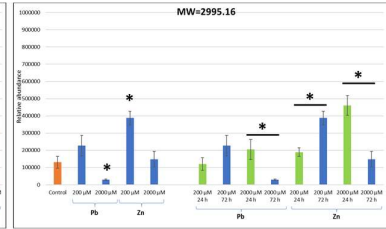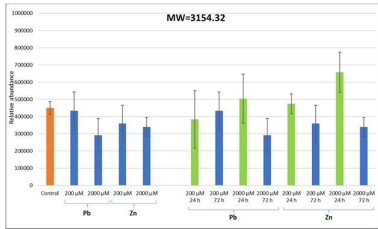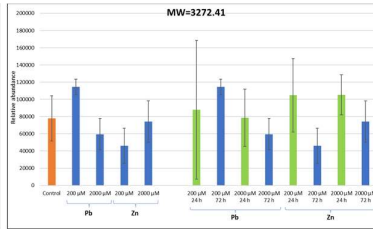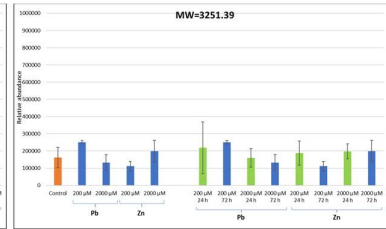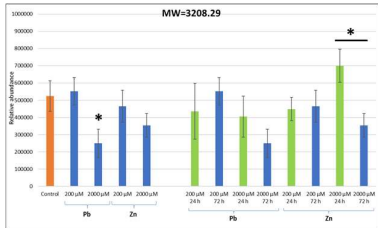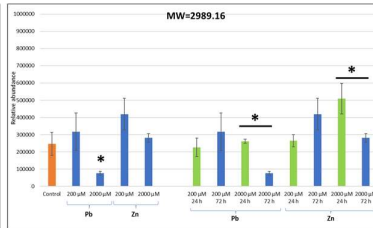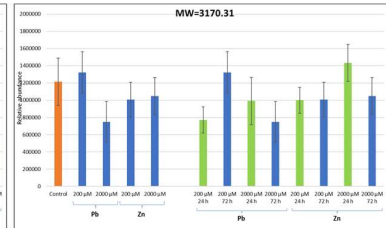

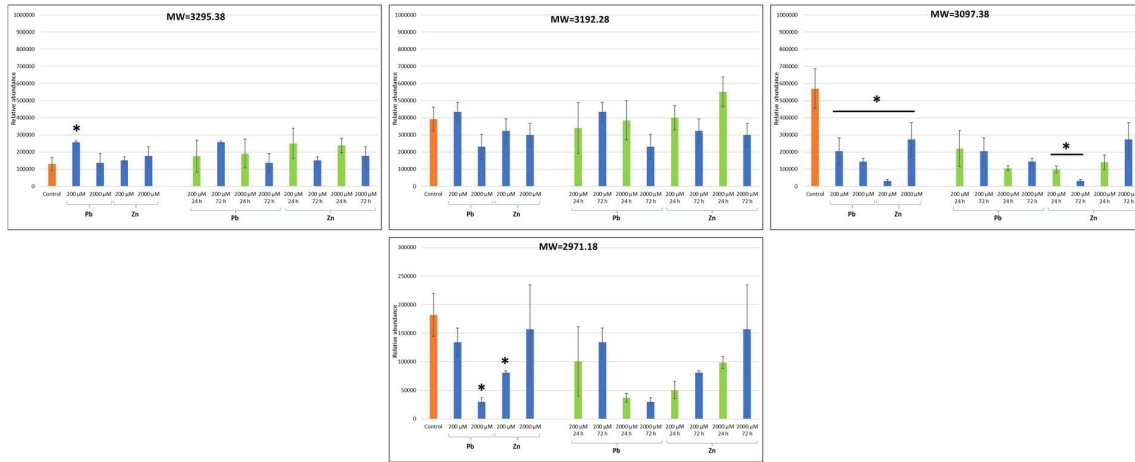

*V. arvensis*:

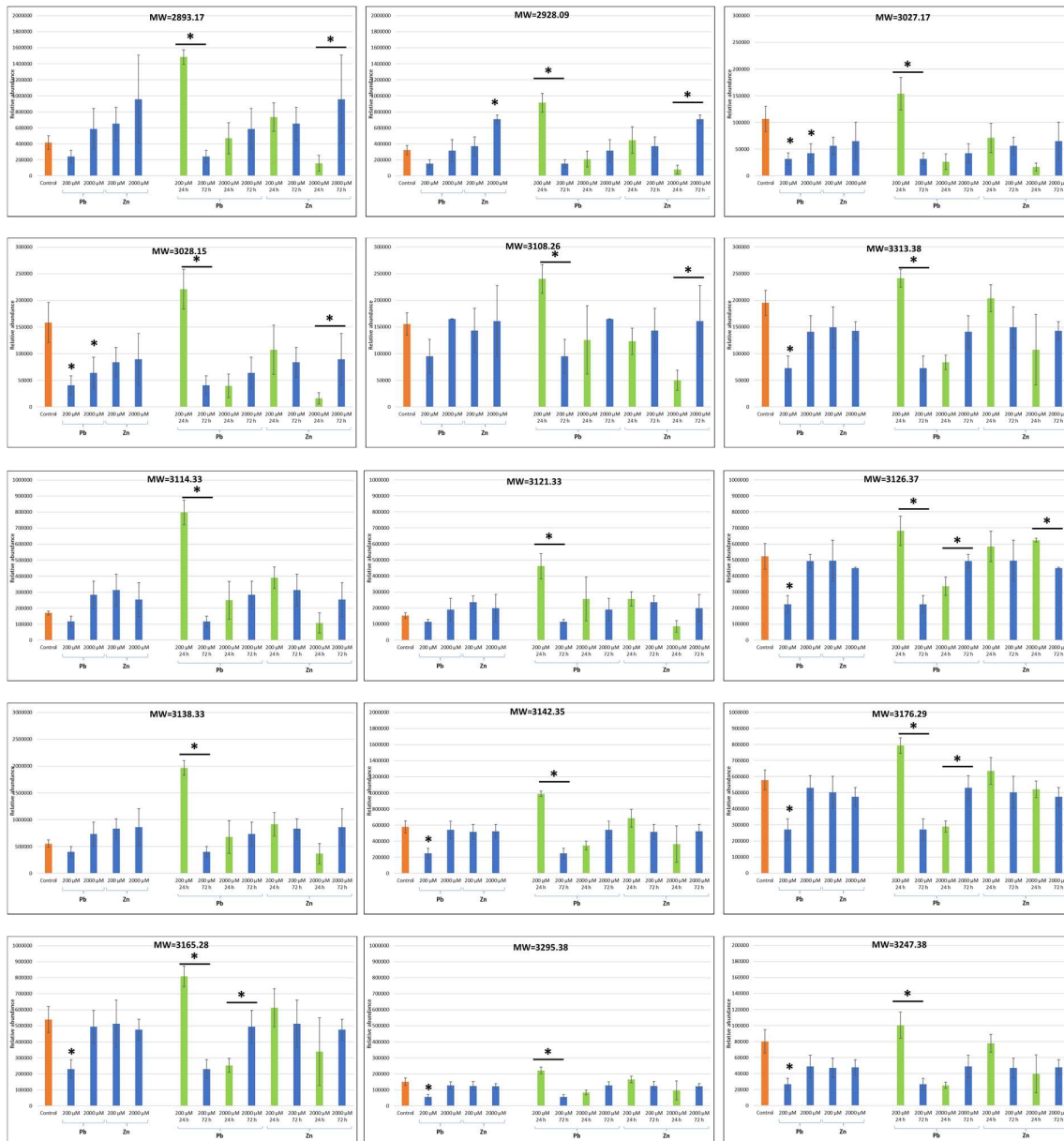

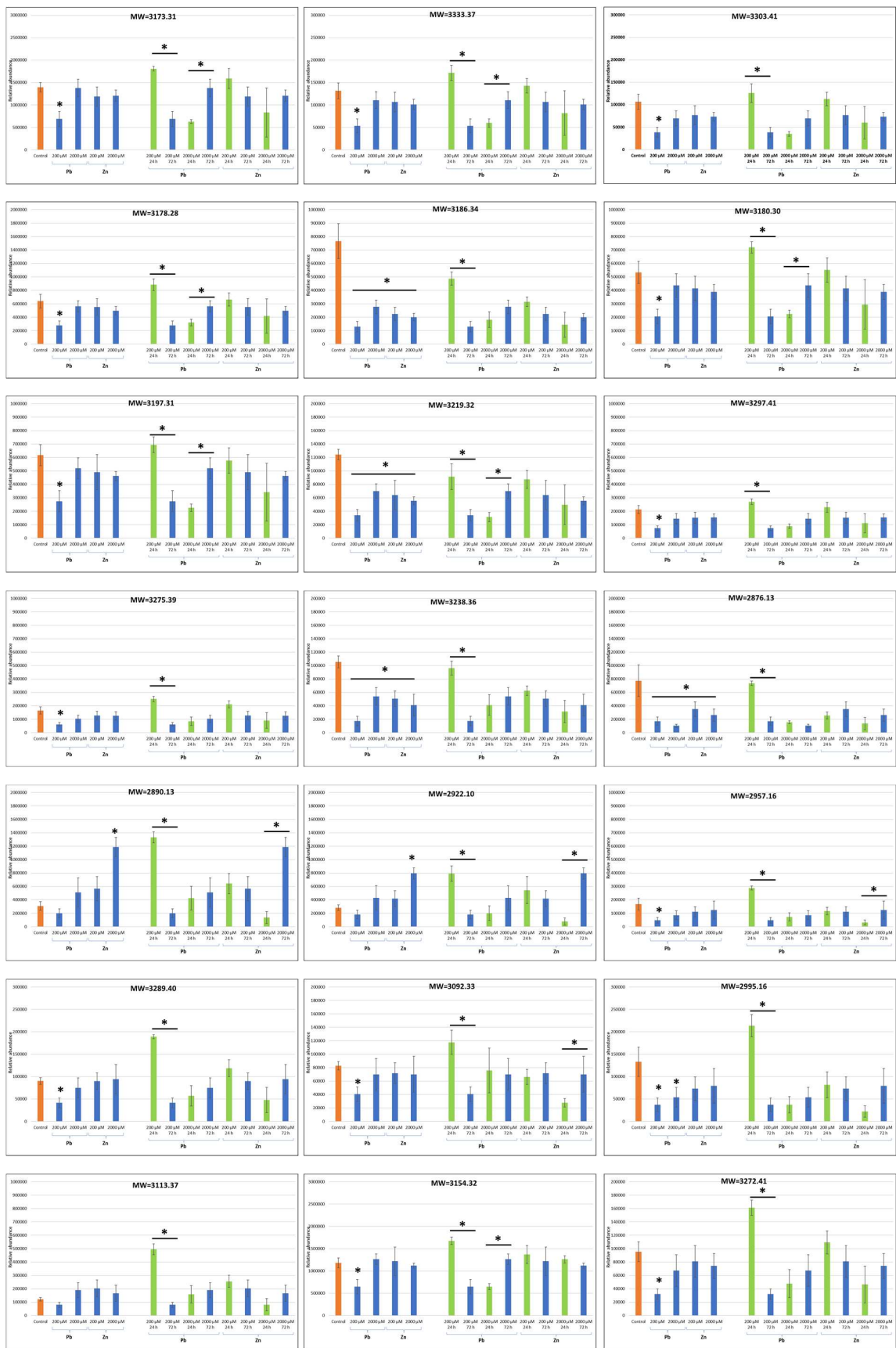

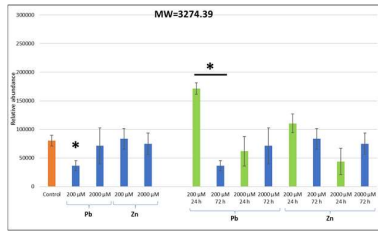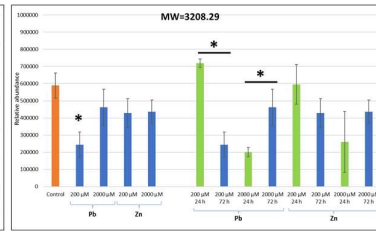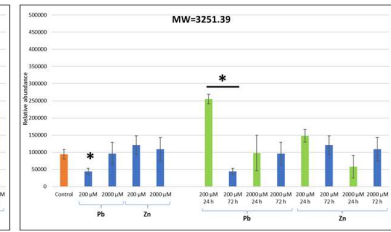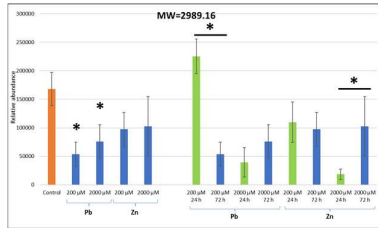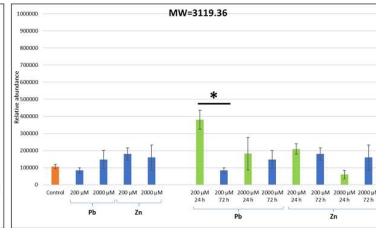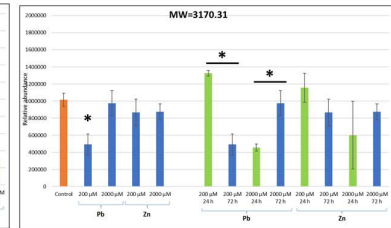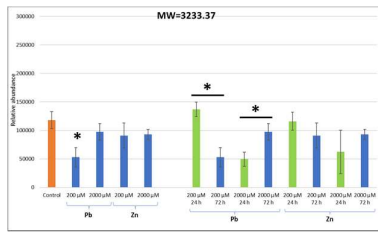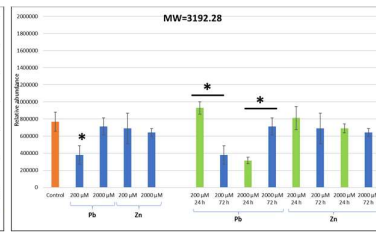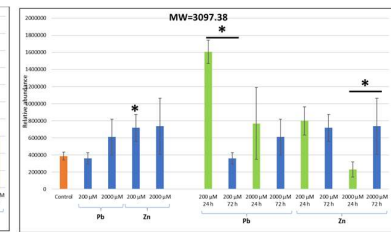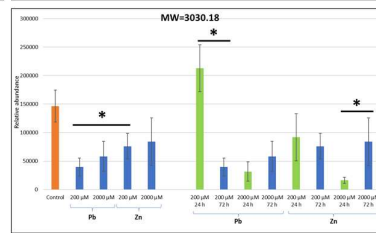

*V. uliginosa*:

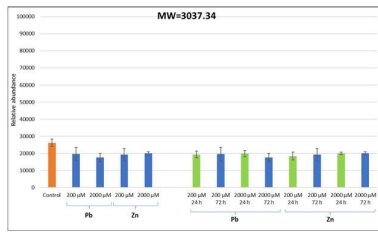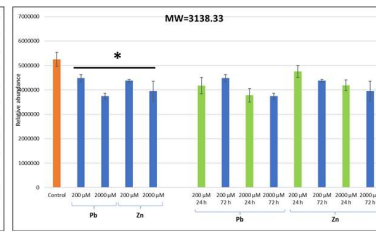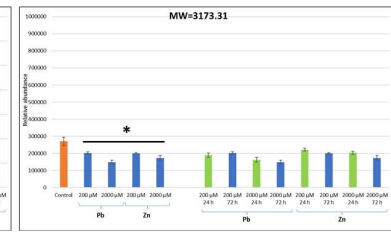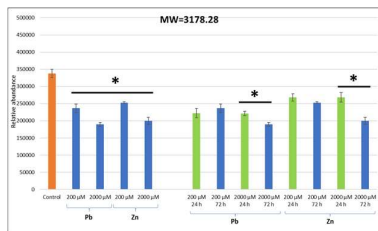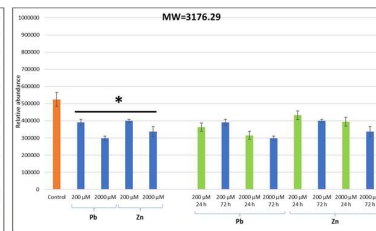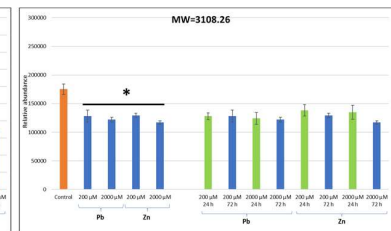

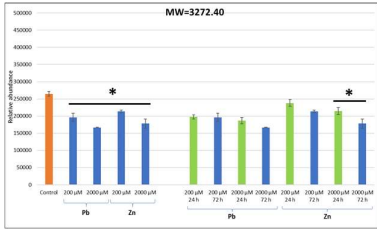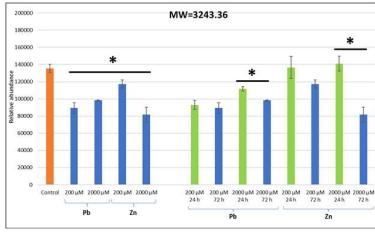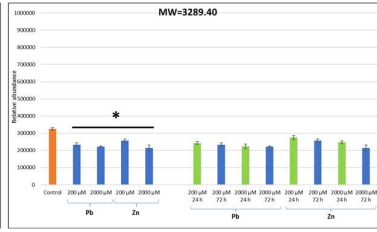

Supplement: Supplementary file 4 — Supplementary Material 2. [file 41598_2024_69018_MOESM4_ESM.pdf]
